# Plant and Animal Protein Sources in Inflammatory Bowel Disease: Current Evidence and Clinical Implications

**DOI:** 10.1002/jgh3.70462

**Published:** 2026-08-27

**Authors:** Mustafa Mohamedrashed, Ayushi Chauhan, Vaios Svolos, Abhinav Vasudevan, Chu K. Yao

**Affiliations:** ^1^ Department of Gastroenterology, Eastern Health Clinical School Monash University & Eastern Health Melbourne Australia; ^2^ Laboratory of Clinical Nutrition and Dietetics, Department of Nutrition and Dietetics, School of Physical Education, Sports Science and Dietetics University of Thessaly Trikala Greece; ^3^ School of Medicine, Dentistry and Nursing, College of Medical, Veterinary and Life Sciences University of Glasgow Glasgow UK; ^4^ Department of Gastroenterology, School of Translational Medicine Monash University & Alfred Health Melbourne Australia

**Keywords:** animal protein, Crohn's disease, diet, inflammatory bowel disease, meat, plant protein, protein, ulcerative colitis, vegetarian

## Abstract

With the consumption of protein consumption increasing globally, there are gaps in our understanding of the differences in protein sources, particularly between animal and plant proteins and their recommendations for patients with Inflammatory Bowel Disease (IBD). This narrative review synthesizes current evidence of how animal and plant proteins differentially exert physiological effects, influence IBD risk, and outcomes. First, protein sources vary in their amino acid profiles, co‐existence with other nutrients and consumption within a dietary pattern, factors that influence their physiological effects. Second, observational data showed possible associations between higher intake of animal protein and risk of Ulcerative Colitis (UC); however, this association is less consistent with Crohn's Disease (CD). High intake of meat, specifically red meat, is also associated with a greater risk for a UC relapse. Third, specific animal proteins are utilized as part of efficacious strategies for the induction and maintenance of CD. In conclusion, the available data suggest that recommendations for the IBD patient should move beyond animal versus plant protein advice but consider individual protein sources and the wider dietary pattern.

## 1 Introduction

Global trends in food consumption patterns have shown a significant rise in protein intake over the past seven decades. A systematic review across 40 countries has shown an increase of 0.5%–0.7% total energy intake from protein (equivalent to ~4–6 g) per decade in regions such as Northern Europe and parts of Asia [1]. This increase has not necessarily been accompanied by an increase in caloric intake. The newly released Dietary Guidelines for Americans reinforce this protein trend, recommending a 1.5–2.0‐fold increase in both animal and plant protein consumption [2]. Accompanying the high protein trends and increasing awareness of food sustainability, there is a shift toward strong consumer demand for plant protein options. Indeed, 40% of national dietary guidelines across the world now incorporate recommendations that promote consumption of plant over animal proteins [3]. Conversely, excessive consumption of animal protein is implicated in the development of chronic gastrointestinal diseases, including inflammatory bowel disease (IBD) [4]. Protein sources, however, differ in their nutritional value and overall health benefits. Therefore, it is important to understand the differences between animal and plant proteins, including whole food sources as well as those that have undergone processing such as exclusive enteral formulas, in the context of IBD to provide clarity in recommendations for both clinicians and patients with IBD. The aim of this narrative review is to explore the evidence for plant and animal protein in the etiology and management of IBD, with a view of providing nuanced and practical recommendations for their consumption. For the purpose of this review, animal protein encompasses red meat, pork, poultry, fish, eggs, and dairy, whereas plant proteins can be derived from legumes, nuts, seeds, soy, grains, and plant‐based meat analogues.

## 2 Methods

A literature search was conducted in PubMed from database inception to April 1, 2026 using combinations of the following keywords: “protein,” “animal protein,” “plant protein,” “red meat,” “meat,” “poultry,” “chicken,” “fish,” “eggs,” “dairy,” “nuts,” “legumes,” “soy,” “fermentation,” “nutritional characteristics,” “digestibility,” “colon,” “diet,” “inflammatory bowel disease,” “ulcerative colitis,” “Crohn's disease,” “colorectal cancer,” and “cardiometabolic health.” English‐language articles relevant to the scope of this narrative review were considered. Additional relevant publications were identified through manual screening of the reference lists of selected articles.

## 3 Protein Sources Vary in Their Physicochemical and Nutritional Characteristics

**FIGURE 1:**
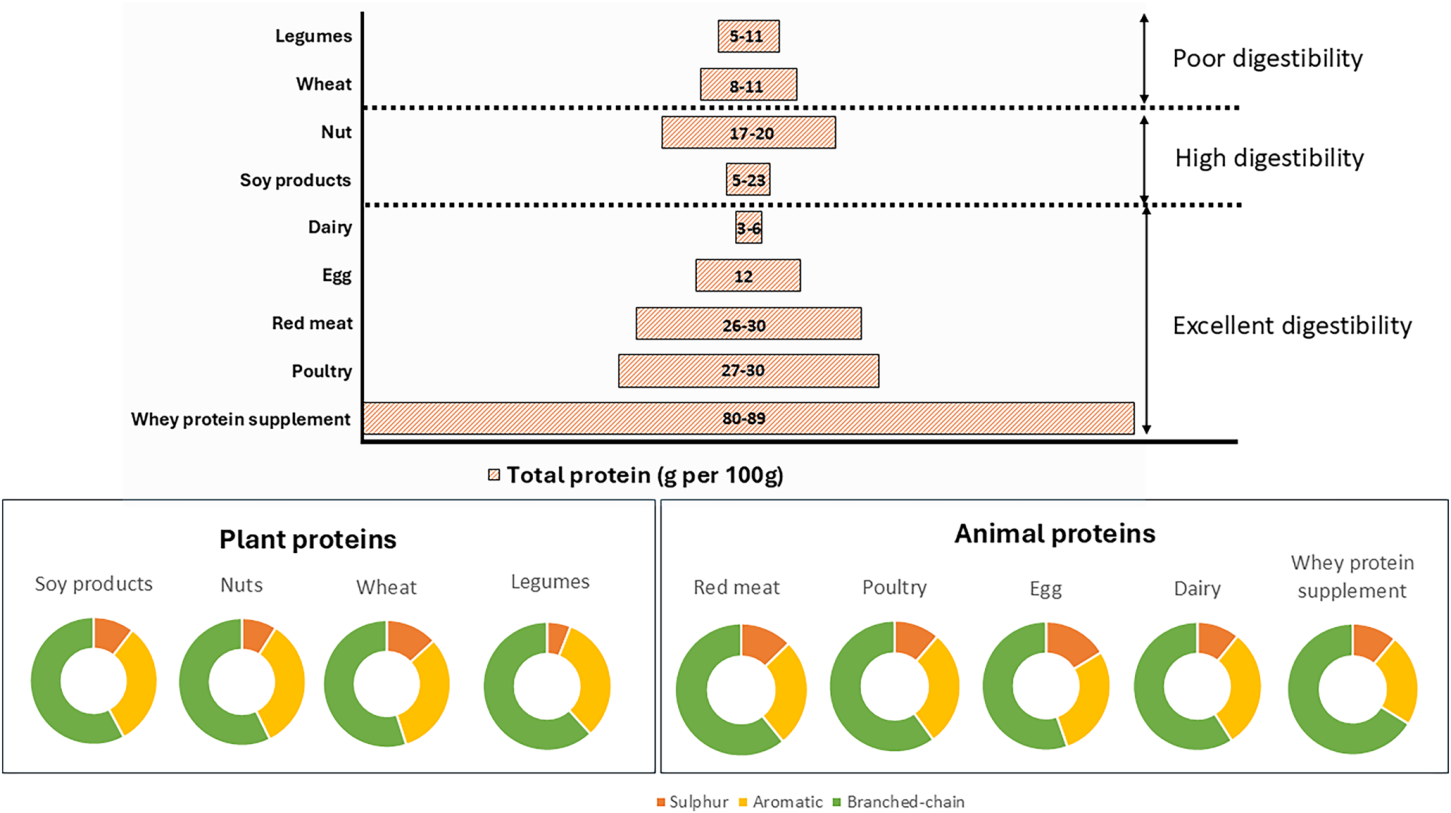
Sources of animal and plant proteins, their extent of digestibility, protein content, and profile of key amino acids that influence the metabolic by‐product of the colonic microbiota. Classification of digestibility was determined by a Digestible Indispensible amino acid score, values for protein and amino acid content were derived from the Australian [5] and United States food composition databases [6].

The quality of animal and plant proteins is often attributed to their amino acid composition. However, other physicochemical properties can also impact digestibility and absorption in the digestive tract, and these differ between animal and plant proteins. For example, as shown in Table 1 and Figure 1, animal proteins are considered good quality proteins due to their high digestibility and provision of all amino acids, despite substantial variations in amino acid profiles across different sources. These amino acid profiles can be classed in several ways, including by the types of metabolites generated during colonic fermentation. For example, red meat, poultry, and egg contain the highest amount of sulfur amino acids [5, 6], a group of amino acids that are fermented by the gut bacteria to produce hydrogen sulphide (H_2_S) and other sulfur metabolites. Comparatively, plant proteins such as wheat and leguminous proteins contain relatively low amounts of sulfur amino acids [5]. A second group of amino acids called aromatic acids—phenylalanine, tryptophan, and tyrosine—are found in high concentrations in poultry, red meat, and nuts. These are fermented to produce indoles and phenols. Thirdly, branched‐chain amino acids, a major group of amino acids, are present in all proteins and are fermented to produce branched‐chain fatty acids (BCFAs).

**TABLE 1:** Summary of differences between animal and plant protein^a^.

Characteristic	Animal protein	Plant protein
Common food sources	Red meat, poultry, fish, eggs, dairy	Legumes, soy, nuts, seeds, grains, plant‐based meat alternatives
Protein content per serving	High protein content (> 10–30 g per serve)	Variable depending on food source (~6–22 g per serve); higher in soy products (e.g., tofu and tempeh) and legumes Larger serves required to achieve similar protein intake as animal protein
Protein quality	Complete proteins containing all essential amino acids	Often lower in one or more essential amino acids Consumption of a variety of plant proteins required to achieve all essential amino acids
Structural characteristics	Less complex protein structure; readily accessible to digestive enzymes	Complex protein matrix with tightly folded mesh structure
Digestibility	Highly digestible (> 90%–95%)	Lower digestibility (resistant proteins) due to structural matrix and presence of digestive enzymes inhibitors
Amino acid profile	Higher in sulfur‐containing amino acids; relatively rich in aromatic amino acids	Amino acid composition varies according to plant source
Co‐existing nutrients	Contains saturated or polyunsaturated fats (omega‐3 in fish), vitamin B12, haem iron, phosphorus. For example: ○ Red meat: saturated fat and haem iron ○ Fish: omega‐3, zinc ○ Egg: saturated, unsaturated (mono‐ and poly‐) fats, phospholipids, and choline ○ Dairy: saturated fat, calcium Devoid of any fiber	Rich source of dietary fiber including resistant starch and oligosaccharides; polyphenols, B vitamins, magnesium, and other phytochemicals
Effects on gut microbiota	A high intake, particularly with low fiber, may favor proteolytic microbes, promote protein fermentation with increased production of nitrogenous metabolites	Fiber‐rich plant foods promote growth of saccharolytic bacteria, greater carbohydrate fermentation and favors production of short‐chain fatty acids Diverts fermentation away from undigested protein
Overall implications for IBD	Evidence varies by source; higher red and processed meat intake has been associated with increased UC risk and relapse, whereas dairy proteins form the basis of effective enteral nutrition therapies	Appear protective, although benefits likely reflect the overall dietary pattern rather than protein source alone

^a^ Information derived from references [7, 8, 9, 10].

The availability of amino acids reaching the colon is also influenced by digestibility and dietary intake, with proteins that are highly digestible resulting in lower amounts of amino acid substrates reaching the colon for microbial fermentation, whereas a higher protein intake increases substrate delivery. Regardless, increased fermentation of animal proteins leads to increased production of nitrogenous metabolites (BCFAs, ammonia, amines, sulfur compounds, phenols, indoles, and N‐nitrosamines) [7]. At high concentrations, these metabolites can exert potentially damaging effects on the colonic epithelium and have been associated with the development of inflammatory lesions in the colon, further discussed in relevance to IBD below [7].

Furthermore, adding to the complexity of plant and animal proteins, both animal and plant proteins exist within a food matrix, which are then consumed within an entire diet. These factors are likely to exert greater influence on their gastrointestinal effects than amino acid profiles alone. First, on a food level, plant proteins have tightly folded “mesh” structures that limit the accessibility of digestive enzymes [8]. Enzyme inhibitors such as amylase trypsin inhibitors present in wheat or soy proteins can further impair the actions of digestive enzymes [8]. Hence, these plant proteins are often termed resistant proteins as their digestion is largely incomplete.

Second, the coexistence of other nutrients with protein can also modulate gastrointestinal effects. For example, fish is not only a rich source of sulfur amino acids but also eicosapentaenoic acid, a polyunsaturated fat which has been shown to inhibit metabolic activities of the colonic microbiota in vitro [9]. Plant proteins also co‐exist with a range of fibers including oligosaccharides, resistant starch and nonstarch polysaccharides (NSP) [11, 12] which can influence the amount of protein escaping digestion but also the fermentative profiles occurring in the colon. In a study of ileostomates, Muir et al. [13] showed that a diet with a moderate animal‐to‐high plant protein ratio, and therefore higher fiber content, increased the excretion of undigested protein compared with a typical Australian diet containing moderate protein and fiber. However, when the types of protein were altered (e.g., replacing animal proteins with soy or a mixture of plant proteins), relatively similar amounts of protein were recovered in the ileal effluent, while substantially greater amounts of resistant starch and NSP became available as substrates for carbohydrate fermentation [13]. Increased delivery of fermentable fiber promotes saccharolytic fermentation in the colon, leading to greater production of short‐chain fatty acids (SCFAs) that are critical for colonic epithelial health and support the growth of beneficial bacteria [14].

Together, these studies suggest that the complexities of animal and plant proteins, including their food matrix, protein source, and co‐existence with other nutrients in the diet, have important implications for their role in modulating their physiological effects in the colon.

## 4 Differential Effects of Protein Sources on the Gut Microbiota

The effects of plant and animal proteins on the gut microbial structure and function have largely been studied in the context of whole diets in healthy individuals. David et al. [14] studied two extremes of animal vs. plant protein intake, with the former replacing all plant foods with animal protein (protein and fat: 30% and 70% total caloric intake, 0 g fiber) and vice versa. Relative to the vegan diet, the carnivore diet showed a significant decrease in beta‐diversity that was not seen in the vegan diet, with a significant increase in several bile‐resistant and proteolytic microbes, including *Bilophila wadsworthia, Alistipes putredinis*, and *Bacteroides* [14]. By‐products of fermentation were also altered in these individuals with an increase in BCFAs that was accompanied by a reduction in major SCFAs, acetate, and butyrate. In a separate study involving 21 561 individuals [15], similar observations in microbial changes were also seen for animal protein intake. Meat consumption was associated with higher abundance of *A. putredinis, Ruminnococcus torques* and *B. wadsworthia*, while Lachnospiraceae, *Butyricicoccus* sp., and *Roseburia hominis* , saccharolytic microbes, and SCFAs producers are associated with the consumption of plant foods. The significance of these microbial signatures for gut health outcomes is, however, unclear. What was interesting is the finding that diets with a high dairy content were associated with a greater abundance of lactic‐acid bacteria, including *Streptococcus thermophilus* and several lactobacillus strains, that may be putatively beneficial for gut health.

It is important to note that the potentially detrimental microbial changes reported by David et al. [14] are only pronounced when dietary modification of animal proteins is extreme, or where intake of animal protein is compromised by lower fiber intake. Supporting this, a randomized controlled trial (RCT) replacing habitual intakes of red meat with either no meat or 600 g/d (~1.3 times the recommended weekly intake), without altering fiber intake, resulted in the production of potentially toxic nitrogenous compounds at levels comparable to carcinogens found in tobacco smoke [16]. In contrast, when background fiber intake is increased, these effects are attenuated. In an RCT of 28 healthy men, doubling protein consumption with animal sources alongside increased fiber did not alter fecal concentrations of protein fermentation metabolites [17]. This likely reflects preferential fermentation of fibers, which also diverts microbes away from fermentation of protein. Taken together, these findings highlight that the totality of what is consumed, namely the ratio of animal to plant protein and background fiber intake, should be considered when critically examining the literature on these proteins.

## 5 Animal Versus Plant Protein: Role in the Etiology of IBD

The rising global incidence of IBD has intensified research into the role of dietary factors in its etiology. In parallel with global trends of increasing protein intake, there is growing interest in the differential roles of animal and plant protein in the development of IBD.

### 5.1 Animal Protein and Risk of IBD

The relationship between animal protein and incidence of IBD is not uniform across both CD and UC as shown in several large prospective studies. One of the first signals linking animal protein to the development of IBD was derived from the French E3N cohort consisting of 67 581 women [4]. A high intake of animal but not plant protein was significantly associated with increased IBD risk (OR: 3.31; 95% CI: 1.41–7.77), and (OR: 3.03; 95% CI: 1.45–6.36) respectively. Among the two IBD subtypes, only the incidence of UC showed a strong dose‐effect association with animal protein intake (*p* = 0.005). Of note, however, the study was underpowered for IBD subtype‐specific risk estimation [4]. In contrast, the European Prospective Investigation into Cancer and Nutrition (EPIC) cohort, which examined the association between diet and dietary patterns among 413 593 participants across 10 countries, animal protein intake was not associated with increased risk of IBD (CD or UC) [18]. The exception was a significant association between the highest vs. lowest quartile of total and red meat intake with the development of UC (HR: 1.45, 95% CI: 1.03–2.04). A more recent and comprehensive meta‐analysis, which pooled 11 cohorts with > 4 million participants and 8000 IBD cases [19] confirmed earlier findings for the lack of association between animal protein consumption and IBD risk (RR: 1.23; 95% CI: 0.81–1.86). However, dose–response analysis showed that each 100 g/day increase in total meat intake was associated with 38% higher IBD risk overall. IBD subtype‐specific analysis demonstrated no significant associations between red or processed meat intake and CD (RR: 1.02; 95% CI: 0.82–1.28 and RR: 1.01; 95% CI: 0.78–1.30, respectively), while modest, nonsignificant trends were observed in UC risk (RR 1.16 for both red and processed meat) [19].

Interestingly, not all animal products carry the same risk profile. Dairy intake was found to be a protective factor in the same study by Talebi et al. [19] with higher dairy consumption being associated with a 19% reduction in IBD risk, potentially due to its putative benefits on the gut microbiota as alluded to earlier. Additionally, the positive associations with dairy were observed for both CD (Relative risk [RR]: 0.69) and UC (RR: 0.84), although this benefit did not persist when adjusted for body mass index (BMI) and alcohol [19]. Furthermore, the EPIC cohort showed that those who consumed milk compared to those that did not had a significantly lower risk of developing CD (OR: 0.30 [95% CI, 0.13–0.65]) [20]. No association was also found for UC with the consumption of low versus high amounts of dairy (OR 0.85, 95% CI, 0.49–1.347) [20]. With respect to other animal protein sources, findings from the E3N cohort indicated that a high egg consumption was not associated with increased IBD risk (HR: 0.97; 95% CI: 0.52–178) [4]. Fish consumption found to be associated with a slight increase in IBD risk (HR:1.83; 95% CI: 1.00–3.36) [4] in one study although this was negated by a much larger analysis of 5 cohort studies (*n* = 4757 cases; RR:1.03; 95% CI: 0.02–1.15) and a nonsignificant trend toward lower risk in CD (RR: 0.92; 95% CI: 0.73–1.15). Finally, poultry consumption had no bearing on IBD risk, for either UC (RR: 0.92; 95% CI: 0.67–1.26) or CD (RR: 1.42; 95% CI: 0.87–2.33).

The proposed mechanism linking specific animal protein to the development of IBD may involve increased protein fermentation and the potentially damaging effects of protein fermentation metabolites, ammonia and phenolic compounds as mentioned above. Increased production of these metabolites can contribute to mucosal injury and trigger inflammatory cascade [5], findings that are mainly derived from in vitro and animal models. Diets rich in animal protein have been found to exacerbate both acute and chronic intestinal inflammation in mice models, although this effect is dependent on the presence of specific gut microbes [21]. Furthermore, most studies are limited by not adjusting for fiber intake, which has implications for understanding whether in the colon and in IBD.

### 5.2 Plant Protein and Risk of IBD

In contrast to animal protein, plant proteins, particularly legumes, nuts, and seeds or those consumed in the context of a plant‐based diet are found to have a neutral or protective relationship with the development of IBD. Recent large‐scale prospective studies from the UK Biobank (*n* = 187 888) and the EPIC cohort (*n* = 341 539) have demonstrated a 25%–29% reduction in the incidence of both CD and UC (HR 0.75; 95% CI: 0.60–0.94; and HR 0.71; 95% CI: 0.59–0.87 respectively) in those adhering to a healthy plant‐based diet (hPDI) [22]. When specific foods were assessed, the Nurses' Health Studies and Health Professionals Follow‐up Study of 223 283 individuals found no associations between nut nor legume intake with CD or UC risk [23]. However, there was a trend toward significance for individuals with a BMI ≥ 25 to have an 86% decrease in risk for CD (95% CI: 0.03–0.56, *p* = 0.06) for every additional serving of nuts consumed per day compared with those with a BMI < 25 [adjusted HR 0.88 (95% CI: 0.45–1.74)] [23].

Moreover, the types of plant protein consumed within a plant‐based dietary pattern carry weight in modification of disease risk. In the UK Biobank and EPIC cohort studies, adherence to unhealthy plant‐based diets (uHPD) comprising refined grains and ultra‐processed plant products may increase the risk of both CD and UC by 48% and 54% (HR 1.48; 95% CI: 1.21–1.82 in UK Biobank and HR 1.54; 95% CI: 1.30–1.84) [22]. It is possible that the reduced fiber intake that is usually associated with consumption of ultra‐processed plant proteins may be the key driver of disease risk, rather than just the level of processing or mere absence of animal protein. Interestingly, these associations were stronger in those with moderate or high genetic susceptibility to IBD [22].

The mechanisms behind the protective benefits of a high intake of plant proteins is likely related to the increased consumption of plant foods as part of a dietary pattern. One of the effects of plant‐based diets as alluded to earlier is the increase in carbohydrate fermentation and the beneficial by‐products generated that mediate protection against gastrointestinal diseases. Increased production of SCFAs, albeit not at excessive levels, for example provides immunomodulatory and antioxidative effects, which strengthen the intestinal barrier and exert anti‐inflammatory effects [24]. Additionally, aside from increased fiber intake with the consumption of plant proteins, other dietary factors such as polyphenols or greater dietary diversity may also mediate beneficial effects on the gut microbiota in ways that confer protection against IBD.

In summary, observations that animal protein is associated with increased risk for developing IBD are inconsistent and further studies adjusting for overall dietary patterns and other confounding factors are needed to better understand the relationship between animal protein sources and disease risk. The role of plant proteins appears to be potentially protective, largely within the context of overall dietary patterns. Importantly, these findings align with the ECCO consensus on dietary management in IBD, which does not issue specific recommendations regarding individual protein sources [25]. Instead, the consensus emphasizes adherence to healthy dietary patterns associated with reduced IBD risk. Such patterns are typically characterized by higher consumption of fruits, vegetables, whole grains, legumes, nuts, seeds, and fish, alongside lower intake of red and processed meats. Therefore, rather than framing plant versus animal protein as a binary opposition, current evidence and guidelines support a more holistic perspective, where the quality and overall composition of the diet take precedence over isolated macronutrient sources.

## 6 Animal Versus Plant Protein: Role in Established IBD

There is emerging evidence that the type and amounts of proteins consumed may play a role in altering disease course. The following sections will examine the evidence first from observational and clinical studies investigating protein sources with risk of developing relapse of IBD, and second, modification of animal or plant proteins in the context of therapeutic diets in active and quiescent IBD.

### 6.1 Animal Protein and Relapse Risk

To date, no studies have specifically examined the consumption of animal protein and risk of relapse for IBD. Instead, studies have mostly focused on meat intake, albeit this was variably defined, with two prospective cohort studies demonstrating a significant correlation between intake of meat with risk of relapse specific to UC. The earliest study was in a small cohort of quiescent UC patients (*n* = 191) which found that consumption of red and processed meat in the top tertile of intake (median 175 g/day) increased the likelihood of a relapse by fivefold (95% CI: 2.1–12.9) compared to those in the lowest tertile of consumption (median 124 g/day) [26]. Of note, red meat intake in this cohort—even in the lowest tertile—is substantially higher than that reported in other populations [27, 28, 29], and may reflect an overestimation of intake with the use of food frequency questionnaires. In the PREdiCCt prospective cohort study of 1091 IBD patients, those with high intake of total (aHR 1.95, 95% CI 1.07–3.56), red (aHR 1.81, 95% CI 1.15–2.84) or white meat (aHR 1.91, 95% CI 1.10–3.30) had a nearly twofold increased risk of developing a UC flare [29]. No significant association was identified for high fish consumption. Conversely, in patients with CD, the PREdiCCt and IBD Partners' prospective cohort studies identified no associations between total or red meat intake and relapse risk (total meat: adjusted HR 0.97 [95% CI: 0.54–1.74]); red meat: OR 1.22 (95% CI 0.75–1.98) over a follow up period of 2 years and 7 months, respectively [29, 30]. The lack of relationship between red meat and CD flares was further supported by an RCT of 213 quiescent CD patients. In this trial, no differences were found in the frequency or time to relapse of those consuming high (≥ 2 servings/week) compared to those with low meat (< 1 serving/week) over the 49 weeks of intervention [31].

Data evaluating other animal protein sources and relapse risk in quiescent IBD are minimal. Only one study evaluating dairy avoidance in IBD patients demonstrated a lack of association with relapse risk for UC over a 1‐year period (OR:0.76; 95% CI: 0.36–1.64) [26]. In the absence of other data, the 2025 ECCO diet consensus therefore recommends reducing intake of red and processed meats in patients with UC for the maintenance of remission [25].

### 6.2 Plant Protein and Relapse Risk

There is scant evidence from epidemiological studies and RCTs to suggest a role of plant proteins in modulating IBD relapse risk. In an observational study of 24 CD patients followed prospectively for 10 years, good adherence to a plant‐based diet in combination with infliximab resulted in a relapse‐free rate of 52% [32]. Similarly, long‐term maintenance data for CD shows a 92% remission rate at 2 years for those following a flexitarian diet (lacto‐ovo‐vegetarian diet with occasional consumption of fish and meat), compared to just 33% on an omnivorous diet (*p* = 0.0003) [33]. However, it is important to note that these studies are derived from a single center's observations and have yet to be formally investigated in a larger RCT to establish robust evidence.

Additionally, studies investigating the role of plant proteins in established IBD have primarily focused on a plant‐based dietary pattern and risk of IBD complications, rather than relapse risk per se. A recent large multinational study in the UK found that high adherence to a hPDI significantly improved disease prognosis, leading to a 50% lower risk of IBD‐related surgery (95% CI: 0.30–0.83) [22]. Conversely, when animal protein was replaced by unhealthy plant‐based proteins, the risk of surgery increased to 112% (95% CI: 1.30–3.44) [22]. This data suggests that while hPDI significantly improves clinical outcomes and reduces surgical risk, the therapeutic benefit is highly dependent on the quality of the plant source, rather than the exclusion of animal protein alone.

### 6.3 Manipulation of Protein Sources as a Key Strategy in Exclusion Diets

#### 6.3.1 Diets That Promote Animal Protein Intake

Manipulation of animal protein intake has emerged as a key strategy for dietary therapies in IBD management. These dietary therapies are summarized in Table 2. However, not all are supported by robust evidence such as a Paleolithic or carnivore diet. Both diets focus on a predominance of animal protein, particularly red meat and exclusion of most carbohydrates. The Paleolithic diet has been explored in a single uncontrolled study of 49 patients with mild–moderate CD and UC with significantly positive results on quality of life and inflammatory markers over a 3‐month period [43]. However, the study was limited by the absence of a control diet, lack of assessment of disease activity scores, and a small sample size. Similarly, while the carnivore diet has gained popularity among IBD patients [44], it remains a “fad” diet as RCT evidence is lacking. The restrictive nature of both diets and its potential carcinogenic risks from negligible intake of dietary fiber compounded by higher heme iron and production of nitrogenous compounds should be carefully evaluated in any patient with IBD considering this as a dietary option.

**TABLE 2:** Summary of dietary therapies in inflammatory bowel disease, predominant protein sources, and clinical evidence.

Dietary therapy	References	Predominant protein source/manipulation	Key protein sources allowed	Highest clinical evidence	Overall efficacy
Exclusive Enteral Nutrition (EEN)	[34]	Milk‐derived formula protein (animal)	Typically whey‐based milk formulas. Soy‐based formulas can be utilized depending on patient's dietary needs	Multiple RCTs and meta‐analyses	First‐line induction therapy in pediatric Crohn's disease; effective in adults
Partial Enteral Nutrition (PEN)	[35]	Milk‐derived formula protein (> 35% total energy intake) and regular diet	Milk‐based formula in combination with regular intake of animal or plant protein	Meta‐analyses and cohort studies	Effective in dose dependent manner for maintenance of remission of CD and when combined with CDED
Crohn's Disease Exclusion Diet (CDED)	[36]	Selective animal proteins with PEN	High intake of chicken and eggs; Fish once weekly. excludes processed foods and additives	High‐quality RCTs	Comparable efficacy to EEN with better tolerability
CD‐TREAT	[37]	Natural animal proteins replacing enteral formula	Lean meat, fish, eggs, lactose‐free dairy, excluding plant proteins	Pilot studies	Promising results in CD; larger RCTs required
Ulcerative Colitis Exclusion Diet (UCED)	[38]	Selective restriction of animal protein	Dairy, chicken, and eggs permitted; red and processed meat restricted	Pilot studies	Promising remission and mucosal healing; requires validation
Milk‐free diet	[39, 40]	Elimination of milk	All protein types except milk	Small randomized trial	Limited and conflicting evidence
4‐SURE diet	[41]	Reduces sulfur‐rich animal proteins	Dairy, eggs, and plant proteins including legumes, nuts, tofu. Limited serving of animal protein including red meat, chicken or fish	Open‐label feasibility study	Clinical and endoscopic improvement in mild‐to‐moderate UC
Mediterranean diet	[42]	Mixed plant and animal proteins	Fish, legumes, nuts, wholegrains moderate dairy; limited red meat	RCTs and observational studies	Improves diet quality and symptoms; beneficial for overall health
Plant‐based diet	[33]	Predominantly plant protein	Legumes, soy, whole grains, vegetables with limited animal foods	Single‐center prospective studies	High remission and relapse‐free survival; external validation required
Paleolithic diet	[43]	Predominantly animal protein	Meat, fish, eggs; excludes grains and legumes	Small uncontrolled study	Insufficient evidence for recommendation
Carnivore diet	[44]	Diet consisting only of animal proteins	All red, processed meats, pork, fish, eggs, dairy	Case series	Not recommended due to lack of evidence and potential nutritional risk

On the other hand, two therapeutic diets with evidence‐base for the induction of CD remission are the Crohn's Disease Exclusion Diet (CDED) and Crohn's Disease Treatment‐With‐Eating Diet (CD‐TREAT). The CDED diet combined with PEN requires daily consumption of two chicken breasts and two eggs along with coverage of 50% calories from dairy based PEN while excluding all pro‐inflammatory foods [45]. Despite its high content of animal protein, the diet has shown efficacy for CD. A recent systematic review and meta‐analysis [46] of 6 RCTs (*n* = 297 CD patients) demonstrated a 2.5‐fold (95% CI: 1.53–4.37) and a 17‐fold greater (95% CI: 1.75–171) overall odds of achieving clinical remission compared to control or steroid therapy respectively. The CDED and PEN combination also resulted in less “rebound” inflammation when patients returned to a regular diet as compared to EEN [36]. Parallel to CDED, a CD‐TREAT diet, designed to mimic the effects of EEN particularly on the intestinal microbiota using solid food [37], includes significant amounts of naturally occurring animal protein such as lean meat, fish, eggs and lactose‐free dairy while excluding plant proteins such as gluten, nuts, seeds, and wholegrains [37]. Preliminary data from the use of the CD‐TREAT diet in an animal model of ileitis and an open‐label trial of five pediatric CD patients demonstrated significant improvements in weighted Pediatric Crohn's Disease Activity Index scores and a marked reduction in fecal calprotectin. Confirmation of these preliminary results through larger, well‐controlled studies is eagerly awaited.

Of note, there is an interesting paradox where observational studies highlighted earlier show a lack of association between specific animal protein and relapse risk in Crohn's disease, yet dietary interventions high in animal protein are effective in inducing remission in patients with Crohn's disease. Several explanations are possible. First, patients with Crohn's disease are at greater risk of increased protein catabolism [47]. Therefore, dietary interventions containing high‐quality proteins may help address nutritional repletion during active disease. Second, animal protein is a rich source of tryptophan. Animal studies have shown that tryptophan metabolites produced by microbial fermentation, including kynurenine, serotonin, and indole derivatives, play a key role in regulating intestinal barrier function [48]. This is further supported by clinical studies of CDED, where alterations in specific tryptophan metabolites are associated with sustained remission on the diet [49]. Together, these findings suggest that the causative dietary agents driving pathogenic mechanisms during active disease are likely to be different from those responsible for relapse.

##### 6.3.1.1 Dairy Protein as a Therapeutic Agent

Exclusive enteral nutrition (EEN) and partial enteral nutrition (PEN) utilize milk‐derived protein as the sole or predominant protein source, replacing all or most solid food as an established therapeutic strategy in the induction and maintenance of remission for CD. This presents a paradox, where animal protein has been linked to inflammatory mechanisms within in vitro and animal model studies; however, a large evidence base supports the use of ultra‐processed milk proteins as the first‐line treatment in pediatric CD, with superior efficacy to oral steroids in the induction of clinical, biochemical, and endoscopic remission [34, 36, 46, 50]. In adults, EEN was not only highly efficacious for the induction of remission in uncomplicated CD [34] but also achieved fistula closure in 75% of CD patients with entero‐cutaneous fistula and remission in 60% of stenosing CD patients [51]. In UC, the efficacy for EEN has been more controversial, with a systematic review concluding limited evidence supporting its use for either induction of remission except in acute severe UC [52]. In one study of ASUC patients, a short EEN course improved corticosteroid responsiveness and reduced hospital stay [53]. This suggests that in specific cohorts of UC, milk protein in EEN may aid in modulating inflammatory pathways during severe flares.

As an alternative to EEN, PEN has demonstrated efficacy in a dose dependent manner, with prevention of relapse in quiescent CD seen when 35%–50% of total energy intake is provided by PEN [35, 54]. Moreover, in patients with loss of response to infliximab, remission rates are significantly improved when PEN is combined with escalated dose infliximab [55]. Currently, PEN provided in amounts < 50% of total energy intake alongside usual diet is not recommended for induction of remission in CD. Although higher volumes are advised, the optimal dose has not been defined [25]. PEN has shown comparable efficacy to EEN when combined with exclusion diets, CDED [35] as alluded to earlier. On the other hand, there is no evidence to support PEN for maintaining remission in UC.

#### 6.3.2 Diets That Restrict Animal Protein

Restrictions of specific animal proteins either exclusively or as part of a multiprong dietary strategy have been investigated for the induction of remission mainly for UC.

##### 6.3.2.1 Dairy Elimination Diets

The initial interest in elimination diets targeting milk as a therapeutic strategy for UC stemmed from observations of patient‐reported symptom resolution on milk elimination diets which then recurred following reintroduction of milk [39]. To date, clinical trials of milk‐free diets in UC have shown mixed results. In one study, 15 patients with quiescent UC randomized to milk‐ or gluten‐free diet with milk for 12 months showed no statistical difference in relapse rates compared to a control group [39, 40]. Similarly, a study in 29 pediatric UC patients failed to show any benefits of a milk elimination diet alongside steroid induction therapy mesalamine on the rate of relapse or clinical response compared to those on a normal diet [56].

There are no studies evaluating a milk‐free diet in CD. Instead, the use of dairy protein as a therapeutic agent in the form of exclusive or PEN has already been discussed [57].

##### 6.3.2.2 Restriction of Animal Protein

There are at least five different published diets that have eliminated or restricted intake of red and processed meats specifically, including various forms of therapeutic diets—“Anti‐inflammatory Diet” [58], UC exclusion diet [38], CDED [36], and the 4‐strategy to a sulphide reducing (4‐SURE) diet [41] for the induction of remission. For example, the UC exclusion diet incorporates restrictions of most animal protein (with the exception of dairy, chicken and egg) with two uncontrolled studies suggesting higher rates of clinical remission and mucosal healing. Separately, in an open‐label feasibility study of a 4‐SURE dietary intervention implemented in 28 patients with mild to moderate UC for 8 weeks, clinical and endoscopic response was 46% and 36%, respectively, suggesting promising clinical efficacy of the diet in controlling inflammation [41]. Further RCT evidence is warranted to understand the clinical efficacy of these diets in UC.

#### 6.3.3 Diets That Promote Plant Protein Intake

In a single clinical trial involving 160 patients with IBD, implementation of a lacto‐ovo‐vegetarian diet together with infliximab therapy achieved a 96% induction of CD remission and a 76% remission rate in severe UC [59]. There are no other studies that have examined dietary therapies that specifically increase plant protein consumption for management of active or quiescent IBD.

## 7 Health Benefits of Animal Versus Plant Proteins Beyond IBD

A dietary pattern that incorporates a wide range of plant proteins has additional health benefits beyond IBD. First, in modulating cardiometabolic health, there is now evidence to show that greater plant protein consumption (> 6% vs. ≤ 3% kcal increments of total energy intake) is significantly associated with a 12% reduction in the risk of cardiovascular mortality (pooled HR 0.88; 95% CI: 0.80–0.96), improvements in blood lipids and pressure, as well as lowered incidence of type 2 diabetes [60]. Conversely, a high intake of animal protein (~14% of total energy intake) is positively associated with increased all‐cause and CVD‐related mortality, especially in those with at least one lifestyle risk factor [61]. Additionally, a 10% increase in total energy intake from animal protein was associated with a 2% higher risk of all‐cause mortality (HR: 1.02; 95% CI: 0.98–1.05) [61]. This increase in mortality risk is further amplified in those with underlying chronic inflammatory conditions like IBD. Mechanistic studies of long‐term red meat intake have shown increased systemic levels of atherogenic metabolites including trimethylamine N‐oxide (TMAO) which are mediated by impaired renal excretion and increased microbial production of TMAO [14, 62]. Excess production of TMAO can impair colonic epithelial integrity and enhance intestinal inflammation [14, 62]. Importantly, not all animal protein sources confer similar CVD and mortality risks. The consumption of fish and poultry appeared to have neutral or even protective effects [61, 63].

Patients with IBD are at an increased risk of developing colorectal cancer (CRC). Red and processed meat, classed as group 2A and group 1 carcinogens by the International Agency for Research on Cancer, are known risk factors for CRC [64]. This classification is based on meta‐analytical evidence of *n* = 26 observational studies where for every 100 g/d increment of red or 50 g/d increment of processed, the RR for CRC is 1.17 (95% CI: 1.05–1.31) and 1.18 (95% CI: 1.10–1.28) respectively [65]. Interestingly, a key “Burden of Proof” study found that while the correlation between unprocessed red meat and CRC risk remains high (RR: 1.17, 95% Uncertainty Index: 1.01–1.78) for every 100 g/d intake, the evidence is weak due to the significant heterogeneity across studies [66]. Conversely, plant‐based diets have been shown to decrease the risk of CRC [67, 68]. In particular, an hPDI had a strong, inverse association with CRC, whereas uPDI was not associated with a reduced risk [68]. Additionally, vegetarians were shown to have a 22% reduced risk of CRC compared to nonvegetarians (HR 0.78; 95% CI: 0.64–0.95), whereas the reduction in CRC risk for pesco‐vegetarians was 43% (HR 0.57; 95% CI: 0.40–0.82) [69].

## 8 Practical Recommendations for the IBD Dietitian and IBD Patient

From the evidence discussed thus far, it is evident that not all protein sources exert similar influences on disease risk or clinical outcomes. Therefore, dietary advice for protein intake in IBD should move beyond a simple “plant versus animal” distinction and instead consider disease phenotype, activity, nutritional status, tolerance, and the wider dietary pattern. Current evidence does not support universal avoidance of animal protein, particularly in CD, where enteral nutrition and exclusion diets may include milk‐derived, egg, poultry, or fish proteins. However, patients with UC in remission should be advised to limit red and processed meat, consistent with relapse‐risk data and guideline recommendations. In the absence of other IBD specific guidance, patients with IBD need to comply with national guidelines and plant proteins should be encouraged within minimally processed, fiber‐rich foods such as legumes, nuts, seeds, soy, and whole grains where tolerated, rather than ultra‐processed plant‐based substitutes. Table 3 summarizes practical recommendations for the consumption of animal and plant protein in IBD.

**TABLE 3:** Summary of practical, evidence‐based recommendations for the consumption and manipulation of animal and plant protein sources in the prevention and management of IBD.

Area	Practical recommendation
Animal protein and IBD risk	High red/processed meat intake may be more relevant to UC risk than CD risk. Avoid framing all animal protein as harmful; consider source, quantity, and dietary context.
Plant protein and IBD risk	Encourage minimally processed plant proteins within fiber‐rich dietary patterns, where tolerated. Healthy plant‐based patterns may be protective for IBD risk; ultra‐processed plant‐based diets may not be beneficial.
Animal protein and relapse risk in IBD	In UC remission, advise limiting red and processed meat; do not routinely restrict all animal protein in IBD.
Diets that promote animal protein	Avoid recommending high animal‐protein diets such as carnivore or paleolithic diet due to lack of evidence and potential risks of nutritional compromise. Use evidence‐based approaches such as EEN, PEN, PEN+CDED in CD with dietitian support. Animal protein can be part of effective therapeutic diets in CD, but benefits may depend on structured dietary strategies, not high animal protein intake alone.
Diets that restrict animal protein	Do not routinely restrict animal protein or dairy in IBD unless within a structured, evidence‐based dietary protocol. Selective reduction of red and processed meat may be considered in UC, with dietitian monitoring to maintain nutritional adequacy, tolerance, and unintended nutritional compromise.
Take‐home message	In the absence of a need for therapeutic diet prescription or specific dietary restrictions, IBD patients should follow general population dietary guidelines. Emphasize balanced, minimally processed diets with adequate protein, fiber, and dietary diversity. In patients with IBD already following a vegetarian diet, encourage consumption of a wide variety of plant proteins to maximize intake of key at‐risk nutrients (e.g., protein, iron, zinc and vitamin B12).

Dietitians should individualize advice, ensure adequate protein quality and energy intake, protect against unnecessary restriction, and support gradual fiber progression during remission while adapting intake during active disease, strictures, or intolerance.

## 9 Conclusion

In conclusion, specific protein sources vary in their nutritional characteristics, effects on the gut microbiota and their role in the development or clinical course of IBD. These characteristics extend beyond the binary classification of animal versus plant protein. Therefore, dietary advice that often demonizes the consumption of all sources of animal protein should carefully consider the current evidence in IBD. Instead, the relationship between different protein sources and IBD risk remains incompletely defined. While observational data demonstrated that higher intake of red and processed meat may be associated with increased risk of UC relapse, these findings were not reproducible in CD. Furthermore, there is a paucity of data examining effects of other nonmeat protein sources and IBD risk, an area that warrants further research. Similarly, evidence supporting plant‐based diet is largely observational and subject to broader confounding factors that are associated with improved dietary patterns or increased fiber intake, limiting the ability to draw firm conclusions. Future research should focus on well‐designed, adequately powered RCTs manipulating the reduction of red or processed meat alone in UC or modifying intakes of specific animal protein as a therapeutic approach in CD to understand the effects of specific proteins with disease outcomes. Mechanistic insights into how these proteins modulate disease pathways are also warranted.

## Funding

Chu K. Yao is a recipient of the National Health & Medical Research Council Emerging Leadership Fellowship (APP 2041054).

## Ethics Statement

The authors have nothing to report.

## Conflicts of Interest

A.V. has served as a speaker for Pfizer and Abbvie. He has been on the advisory board for Pfizer, Abbvie, and Ferring. C.K.Y. has received travel honoraria from Sandoz, Viatris, and Yakult Australia. The other authors declare no conflicts of interest.

## Data Availability

Data sharing not applicable to this article as no datasets were generated or analysed during the current study.
